# Establishment and Characterization of a Novel Head and Neck Squamous Cell Carcinoma Cell Line USC-HN1

**DOI:** 10.1186/1758-3284-2-5

**Published:** 2010-02-22

**Authors:** Daniel J Liebertz, Melissa G Lechner, Rizwan Masood, Uttam K Sinha, Jing Han, Raj K Puri, Adrian J Correa, Alan L Epstein

**Affiliations:** 1 Department of Pathology, Keck School of Medicine of the University of Southern California, Los Angeles, CA 90033, USA; 2 Department of Otolaryngology, Keck School of Medicine of the University of Southern California, Los Angeles, CA 90033, USA; 3 Tumor Vaccines and Biotechnology Branch, Division of Cellular and Gene Therapies, Center for Biologics Evaluation and Research, Food and Drug Administration, Bethesda, MD 20892, USA

## Abstract

**Background:**

Head and neck squamous cell carcinoma (HNSCC) is an aggressive and lethal malignancy. Publically available cell lines are mostly of lingual origin, or have not been carefully characterized. Detailed characterization of novel HNSCC cell lines is needed in order to provide researchers a concrete keystone on which to build their investigations.

**Methods:**

The USC-HN1 cell line was established from a primary maxillary HNSCC biopsy explant in tissue culture. The immortalized cells were then further characterized by heterotransplantation in Nude mice; immunohistochemical staining for relevant HNSCC biomarkers; flow cytometry for surface markers; cytogenetic karyotypic analysis; human papillomavirus and Epstein-Barr virus screening; qRT-PCR for oncogene and cytokine analysis; investigation of activated, cleaved *Notch1 *levels; and detailed 35,000 gene microarray analysis.

**Results:**

Characterization experiments confirmed the human HNSCC origin of USC-HN1, including a phenotype similar to the original tumor. Viral screening revealed no HPV or EBV infection, while western blotting displayed significant upregulation of activated, cleaved *Notch1*.

**Conclusions:**

USC-HN1, a novel immortalized cell line has been derived from a maxillary HNSCC. Characterization studies have shown that the cell line is of HNSCC origin and displays many of the same markers previously reported in the literature. USC-HN1 is available for public research and will further the investigation of HNSCC and the development of new therapeutic modalities.

## Background

**Figure 1:**
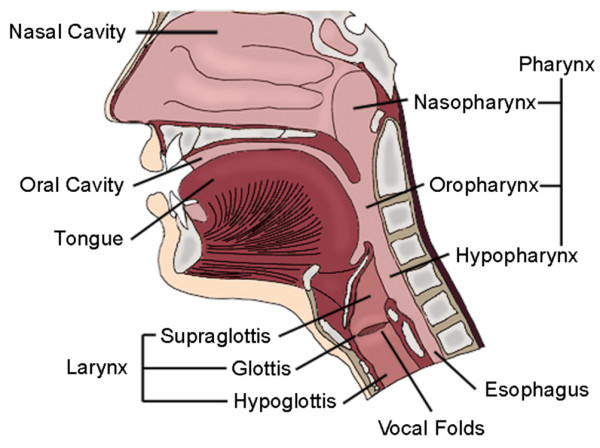
**Schematic of the upper aerodigestive tract and locations of various head and neck malignancies**.

Head and neck squamous cell carcinoma (HNSCC) represents a cancer of increasing incidence worldwide with more than 45,000 head and neck malignancies diagnosed each year, of which greater than 90% are of squamous cell origin. This particularly lethal cancer, the sixth most common world-wide, has not seen an improvement in overall survival in more than four decades [1,2]. Standard-of-care treatment for the disease has been limited to surgical resection or combination chemotherapy and radiation therapy. Despite these treatments, the high rates of primary-site recurrence and common metastases to loco-regional lymph nodes are responsible for the dismal prognosis of HNSCC. Clinically, more than one half of patients with loco-regional advanced disease treated with chemoradiation, surgery or both experience recurrence within two years [3-5]. The presence of lymph node metastases alone decreases the chances of long-term survival by 50% [4]. Bio-molecular research into the cause of HNSCC has had some success; however, without the ongoing development of newly-established HNSCC cell lines, researchers are limited in these pursuits. At the present time, most of the currently available HNSCC cell lines deposited at the American Type Tissue Collection (ATCC) are derived from lingual tumors [1] despite the fact that there are multiple anatomically-exclusive locations from which HNSCC can develop. As shown in Figure 1, HNSCC tumors can arise from any location of the upper aerodigestive tract, including the nasal cavity, sinus cavities, oral cavity, pharynx, or larynx. The various locations associated with malignant transformation implore the need for a wide-ranging database of tumor cell lines representative of all of the anatomic locations. Secondly, distinct biomodels of HNSCC have been established based on the viral infectivity and carcinogenic exposure of the patient. By establishing cell lines representative of the entire upper aerodigestive tract, a comprehensive database would be available to elucidate the development and progression of HNSCC. Moreover, these types of studies could lead to the discovery and advancement of targeted therapies that might alter the clinical outcome of these tumors.

Current research has delineated many generalized and specific markers to characterize HNSCC cell lines. Histologically, HNSCC is a squamous epithelial carcinoma with variable degrees of keratinization. Well-differentiated cell lines may display keratin pearls, whereas poorly differentiated, anaplastic cell lines may have little-to-no keratin production. HNSCC is typically characterized by a malignant phenotype including large, pleomorphic nuclei and large or multiple nucleoli; cytoplasmic vacuolation with abundant cytoplasm; intercellular bridging; and high numbers of mitotic figures, both typical and atypical. Beside these morphologic features, surface and intracellular markers are also used to identify the cell line lineage. Along with traditional markers such as FABP5, epidermal growth factor receptor, E-cadherin, CD74, and CD24, newly published biomarkers for the staining of HNSCC primary tumor biopsies include IL13Rα2, CD44v6, and the stem cell marker CD133 [6]. The population of cancer stem cells (CSC) within the tumor biopsy represented by CD44^+^CD133^+ ^cells has been shown to have a high incidence of metastasis and invasion. These cells, however, have been found to be a rare subset of cells (2-10%) in the overall tumor cell population [7,8]. Karyotypic analysis of HNSCC typically reveals an aberrant chromosome set with various deletions, translocations, and double chromosomes, including a deletion of the small arm of chromosome 3 found in many epithelial cancers [9]. Human papilloma virus (HPV), specifically HPV subtype-16 is associated with HNSCC in about 30% of cases and is most commonly associated with tumors arising from the oropharynx. HPV-positive HNSCC is now considered a distinct biomodel from HPV-negative HNSCC, occurring in patients without the usual history of alcohol and/or tobacco use [10]. Epstein-Barr virus (EBV) is a commonly-associated Herpes virus linked with nasopharyngeal carcinoma not found in HNSCC. Bergmann *et al*. [3] have reported extensive evidence of the immunomodulatory effects of HNSCC, including the local and regional suppression of the immune system by interleukins, TGF-β, and other cytokines. Oncogenes such as *c-myc*, and *c-Kit *and mutations in tumor suppressor genes p53 and Rb are also characteristically found in HNSCC [4,11-13]. Lastly, it has been reported that *Notch1*, an embryonically-associated receptor for inhibition of differentiation, may play a role in the oncogenesis of multiple types of cancers including leukemias, lung, melanoma, breast, and neurological tumors [14] but its role in HNSCC has not been studied extensively to date.

In this report, we now describe the establishment and characterization of a unique HNSCC cell line designated USC-HN1. The cell line was derived from an invasive primary right superior alveolar ridge squamous cell carcinoma in a nonsmoking patient (Stage IVa, T4aN0M0, based on the American Joint Commission on Cancer Staging, 6^th ^Ed.) and has been found to recapitulate the phenotype of the original tumor biopsy. Heterotransplantation and cytogenetic studies demonstrate its oncogenic derivation and monoclonality, respectively. This cell line has been made available for others in the scientific community through the American Tissue-type Cell Collection (ATCC, http://www.atcc.org) and represents an important model for further studies of HNSCC.

## Methods

### Cell Lines and Cells

HeLa, HUT102, Raji, FaDu, and SW579 cell lines were obtained from the ATCC. All cell lines were maintained in complete medium in humidified incubators at 37°C with 5% CO_2_. IRB approval from the USC Keck School of Medicine (HS-09-00048) has been obtained for the collection and use of HNSCC tumor biopsies. Tumor biopsies were surgically resected and placed into 50 mL collection tubes containing approximately 30 mL of RPMI-1640 medium with 20% FCS, 1% Antibiotic-Antimycotic Solution (Mediatech, Inc., Manassas, VA), 10 ug/ml Ciprofloxacin-HCl (Mediatech, Inc.), and 10 ug/ml Gentamicin Sulfate (Irvine Scientific, Santa Ana, CA). The tubes were immediately put on ice and transported to the laboratory for tissue dissociation.

### Establishment of Cell Line USC-HN1

Tumor biopsies were mechanically dissociated into small tissue fragments using scissors and forceps. Fragments were then further dissociated enzymatically in 20 ml of RPMI-1640 medium containing 10% FCS, and 0.2 micron membrane filtered 0.01% hyaluronidase, 0.1% collagenase, and 0.01% DNase (Sigma Chemical Co., St. Louis, MO) for 40 min in a 37°C water bath with intermittent mixing. After digestion, the cells and fragments were washed once in complete medium (RPMI-1640 medium containing 20% FCS, 1% Antibiotic-Antimycotic Solution, 10 ug/ml ciprofloxacin HCl, and 10 μg/ml Gentamicin Sulfate), treated for 30 seconds in 30 ml sterile filtered RBC lysis buffer (8.3 g NH_4_Cl, 1 g KHCO_3_, and 0.037 g EDTA/L dH_2_O), washed again in complete medium, and seeded in two T-75 flasks. After 2-3 days of incubation in a humidified 5% CO_2 _37°C incubator, the fragments were repeatedly pipetted with a 2 ml glass pipette for further dissociation. After centrifugation, the cells and residual small fragments were resuspended in freezing medium (RPMI-1640 medium with 30% FCS, 1% Penicillin/Streptomycin, and 10% DMSO), aliquoted into four-six 1.8 ml cryotubes (Nunc, Denmark), and placed in liquid nitrogen for long-term storage.

Cells and fragments that adhered to the T-75 flasks were grown in complete medium for 2 weeks before being trypsinized and passaged weekly to new flasks. When the malignant cells were seen to grow, the normal fibroblastic cells were removed by differential trypsinization to enrich the malignant cell population. Finally, malignant cells were cloned in petri dishes using cloning rings to isolate a pure population of HNSCC cells to establish the cell line. The doubling time was determined by cell count measurements at 24 hr intervals for one week from cells in culture after trypsinization. After establishment of the cell line, interval screening for Mycoplasma was performed using the MycoAlert Mycoplasma Detection Kit (Lonza, Rockland, ME).

### Heterotransplantation in Nude mice

Six-week-old female Nude mice were purchased from Harlan Sprague Dawley (Indianapolis, IN). Institutional Animal Care and Use Committee-approved protocols and institutional guidelines for the proper humane care and use of animals in research were followed. Mice (n = 5) were injected s.c. in the flank with a 0.2 mL inoculum of 5 × 10^6 ^viable USC-HN1 cells. Three weeks after implantation, tumors were removed and the tissue was either flash frozen in liquid nitrogen or fixed in 10% neutral buffered formalin overnight at room temperature for paraffin-embedded procedures.

### Immunohistochemistry

For immunohistochemistry (IHC) studies, cytospin preparations of USC-HN1 cells and tissue sections of USC-HN1 tumors grown in Nude mice were used and compared to stained, fixed slides of the original tumor. USC-HN1 cells grown for 24 hr directly on sterile printed 25 × 75 mm glass slides (Bellco Glass, Inc., Vineland, NJ) were fixed sequentially with 2% parafolmaldehyde (Polysciences, Inc., Warrington, PA) for 10 min at room temperature and acetone for 5 min at -20°C. For tissue sections, excised heterotransplanted USC-HN1 tumors from Nude mice were fixed overnight in 10% neutral buffered formalin and embedded in paraffin blocks. For cell culture and tissue morphology studies, Wright-Giemsa and hematoxilin & eosin stains were used, respectively on air-dried cytospin preparations. In addition, USC-HN1 cytospin preparations and 5 micron tissue sections were stained with monoclonal antibodies against human CD44 (clone DF1485 Dako Corp., Carpinteria, CA), E-cadherin (clone 4A2C7 Invitrogen, Carlsbad, CA), epidermal growth factor receptor (EGFr) (clone E30 Biogenex, San Ramon, CA), keratin (clone AE1/AE-3 Covance, Berkeley, CA), p53 (clone 1801 CalBiochem, San Diego, CA), and Rb (clone RbG3-245 BD Biosciences, San Diego, CA). Observation, evaluation and image acquisition were made using Leica DM2500 microscope (Leica Microsystems, http://www.leica-microsystems.com) connected to an automated, digital SPOT RTke camera and SPOT Advanced Software (SPOT Diagnostic Instrument Inc., http://www.diaginc.com). Images were further resized and brightened for publication using Adobe Photoshop software (Adobe, http://www.adobe.com).

### Flow cytometry

Single cell suspensions (1 × 10^6 ^cells in 100 μl) in FACS buffer (1% FCS in PBS) were stained with FITC, PE, PerCPCy5.5, and APC conjugated antibodies. For intracellular staining, cell surface staining was performed first, followed by buffer fixation/permeabilization (eBioscience, San Diego, CA) and intracellular staining. Antibodies used were: CD24 (ML5), CD74 (M-B741), E-cadherin (36/E-cadherin) (BD Biosciences); IL-13Rα (B-D13), c-Kit (104D2) (Santa Cruz Biotechnology, Santa Cruz, CA); CD44v6 (VFF-7) (Abcam, Cambridge, MA); CD133 (TMP4) (eBioscience); FABP5 (311215) (R&D Systems, Minneapolis, MN); and anti-human Epidermal Growth Factor Receptor (CTL-R2) (Cancer Therapeutics Laboratories, Inc., Los Angeles, CA). Staining with isotype controls antibodies (eBioscience) was performed in parallel, and all samples were done in duplicate. Samples were run on a FACS Calibur flow cytometer (BD) and data acquisition and analysis were performed using Cell Quest Pro software (BD) at the USC Flow Cytometry core facility.

### Cytogenetics

Karyotype analysis was performed by the Division of Anatomic Pathology, City of Hope (Duarte, CA) using cultured USC-HN1 cells.

### Polymerase chain reaction (PCR) viral screen

Genomic DNA was isolated from USC-HN1, HeLa, HUT102, Raji, FaDu, and SW579 cells using TRIreagent (Sigma) per manufacturer's instructions. For PCR, 50-100 ng of DNA was amplified with specified primers (300 nM final concentration) using REDTaq ReadyMix PCR Master Mix (Sigma) in a 25 μl reaction and run on an iCycler (BioRad, Hercules, CA). HPV infectivity was screened using previously reported consensus primers MY09/MY11 (expected product ~450 bp) and GP5+/GP6+ (expected product ~150 bp) [15,16]. Consensus primers for the EBV gene EBNA2 (expected product ~600 bp) was used to screen for the presence of EBV as previously reported [17].

### Cytokine and oncogene analysis by qRT-PCR

Total RNA was isolated from USC-HN1 and FaDu human pharyngeal carcinoma cell lines by RNeasy Mini Kit (Qiagen, Valencia, CA) per manufacturer's instructions. RNA was DNase treated using Turbo DNase (Applied Biosciences, Foster City, CA) per manufacturer's instructions. For qRT-PCR, 100-200 ng of DNase-treated RNA was amplified with Power SYBR Green RNA-to-CT 1-Step Kit (Applied Biosciences). Primer sequences for cytokines and oncogenes were from the NIH qRT-PCR database [[18], http://primerdepot.nci.nih.gov] and were synthesized by the USC Core Facility (Table 1). Specific markers analyzed included p53, Rb, *c-myc*, *c-Kit*, VEGF-A, VEGF-C, Cox2, TGFβ1, TGFβ2, IL-1β, IL-4, IL-6, IL-8, and IL-10. For amplification, samples were run on a Stratagene Mx3000P cycler with MxP QPCR software (Strategene, La Jolla, CA). Gene-specific amplification was normalized to GAPDH and fold change in gene expression calculated relative to Universal Human Reference RNA (Stratagene). For statistical analysis, the student's t-test for independent samples was used with a significance level α = 0.05 to compare gene expression between samples. Statistical tests were performed using GraphPad Prism software (La Jolla, CA).

**Table 1:** Cytokine and Oncogene Primers for qRT-PCR

Target	Forward Primer	Reverse Primer
p53	5' - GCTCGACGCTAGGATCTGAC - 5'	5' - CAGGTAGCTGCTGGGCTC - 3'
Rb	5' - GTTGGTCCTTCTCGGTCCTT - 3'	5' - CAAAGCAGAAGGCAACTTGA - 3'
c-myc	5' - CTCCTCCTCGTCGCAGTAGA - 3'	5' - GCTGCTTAGACGCTGGATTT - 3'
c-Kit	5' - GCCCACGCGGACTATTAAGT - 3'	5' - CTGGGATTTTCTCTGCGTTC - 3'
VEGF-A	5' - CACACAGGATGGCTTGAAGA - 3'	5' - AGGGCAGAATCATCACGAAG - 3'
VEGF-C	5' - CTCCAGATCTTTGCTTGCAT - 3'	5' - CTGTGGCGTGTTCTCTGCT - 3'
COX2	5' - TTCAAATGAGATTGTGGGAAAATTGCT - 3'	5' - AGATCATCTCTGCCTGAGTATCTT - 3'
TGFβ-1	5' - GCAGAAGTTGGCATGGTAGC - 3'	5' - CCCTGGACACCAACTATTGC - 3'
TGFβ-2	5' - CTCCATTGCTGAGACGTCAA - 3'	5' - CGACGAAGAGTACTACGCCA - 3'
IL-1β	5' - GGAGATTCGTAGCTGGATGC - 3'	5' - GAGCTCGCCAGTGAAATGAT - 3'
IL-4	5' - AGCGAGTGTCCTTCTCATGG - 3'	5' - CAGCCTCACAGAGCAGAAGA - 3'
IL-6	5' - CATTTGTGGTTGGGTCAGG - 3'	5' - AGTGAGGAACAAGCCAGAGC - 3'
IL-8	5' - AGCACTCCTTGGCAAAACTG - 3'	5' - CAAGAGCCAGGAAGAAACCA - 3'
IL-10	5' - GCCACCCTGATGTCTCAGTT - 3'	5' - GTGGAGCAGGTGAAGAATGC - 3'
GAPDH	5' - CTCTGCTCCTCCTGTTCGAC - 3'	5' - TTAAAAGCAGCCCTGGTGAC - 3'

### Western blot for activated *Notch1*

For western blots, 50 μg of protein from sonicated whole cell lysates were fractionated in a 10% Tris-glycine polyacrylamide gel, electrotransferred to PDVF membranes, and probed overnight with primary antibody for activated *Notch1 *(clone Val1744) (Cell Signaling, Danvers, MA). Blots were stripped and reprobed for GAPDH (clone FL-335) (Santa Cruz Biotech), to normalize the amount of sample loaded. Horseradish peroxidase-conjugated secondary antibodies (Caltag, Burlingame, CA) were then applied, followed by signal detection using Immobilon Western Chemiluminescent HRP Substrate (Millipore, Billerica, MA).

### Microarray gene expression profiling

Total RNA from USC-HN1 was collected and analyzed by microarray as previously described [6]. Briefly, 5 μg of total RNA was reverse-transcribed using 5'-amino-modified primers with amino-allyl-dUTP. cDNA synthesized from USC-HN1 cells was labeled with Cy5 dye, and cDNA from universal RNA (uRNA) labelled with Cy3 dye. Labeled and combined cDNA probes were denatured, mixed in SlideHyb #1 hybridization buffer (Ambion, Austin, TX), and placed onto microarray slides. Thirty five thousand oligonucleotide arrays were hybridized at 42°C in MAUI hybridization system (BioMicro Systems) for overnight, washed with 1 × SSC with 0.05% SDS and 0.1 × SSC buffers, and then were quickly spin-dried. Microarray slides were scanned on a GenePix 4000B scanner (Axon Instruments, Inc., Foster City, CA) with a 5 μm resolution. Scanned raw images were analyzed and data files were generated with GenePix Pro 5.1 (Axon) software. For analysis, data files were uploaded into mAdb (microarray database), and analyzed by the software tools provided by the Center for Information Technology (CIT), NIH. A standard global normalization approach was used for each experiment. All of the extracted data was normalized using a 50^th ^percentile (median) normalization method. Statistical analyses and *t*-test were performed to identify differentially expressed genes.

## Results

### Case report of patient NR with Invasive Right Maxillary SCC

**Figure 2:**
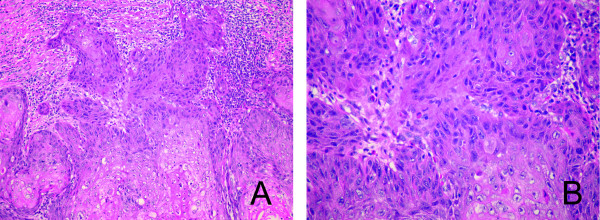
**Hematoxilin and eosin stained histologic sections from the original tumor**. An invasive well-differentiated squamous cell carcinoma, the tumor is arranged as cohesive islands of cells showing nuclear pleomorphism with occasionally prominent nucleoli. Mitotic activity is present including atypical forms. In areas, abundant keratin production is present including whirling "keratin pearl" configurations. Intercellular bridges are visible at high power magnification. Surrounding the invasive tumor, a dense lymphoplasmacytic inflammatory infiltrate is present (A ×200, B ×400 original magnification).

Patient NR is a 57-year-old Hispanic female with a past medical history of hypothyroidism and dyslipidemia. Of note, she does not have a family history of head and neck cancer nor does she have a history of tobacco or alcohol use. In February, 2009 she presented to her primary care physician with a three-month history of fatigue, weight loss, right-sided facial pain, oral ulcers, loose teeth, and bleeding gingiva. A right anterior maxillary gingival biopsy subsequently revealed a well-differentiated squamous cell carcinoma. A series of CTs, including the sinuses and neck, were performed which showed an extensive mass of the right hard and soft palates extending into the right maxillary sinus with significant bone destruction without noted nodal metastases (Stage IVa, T4aN0M0). In May, 2009 the patient underwent a complex resection and reconstruction procedure including a complete right maxillectomy and ethmoidectomy with a split-thickness skin graft repair. Intraoperative frozen section margins of the maxillary crest, pterygoid plates, palate, ethmoid air cells, and buccal cavity were all clear and free of tumor. Final pathological diagnosis of the resected right maxilla indicated an invasive, well-differentiated squamous cell carcinoma, keratinizing type with perineural invasion (Figure 2).

### Establishment of the USC-HN1 cell line

**Figure 3:**
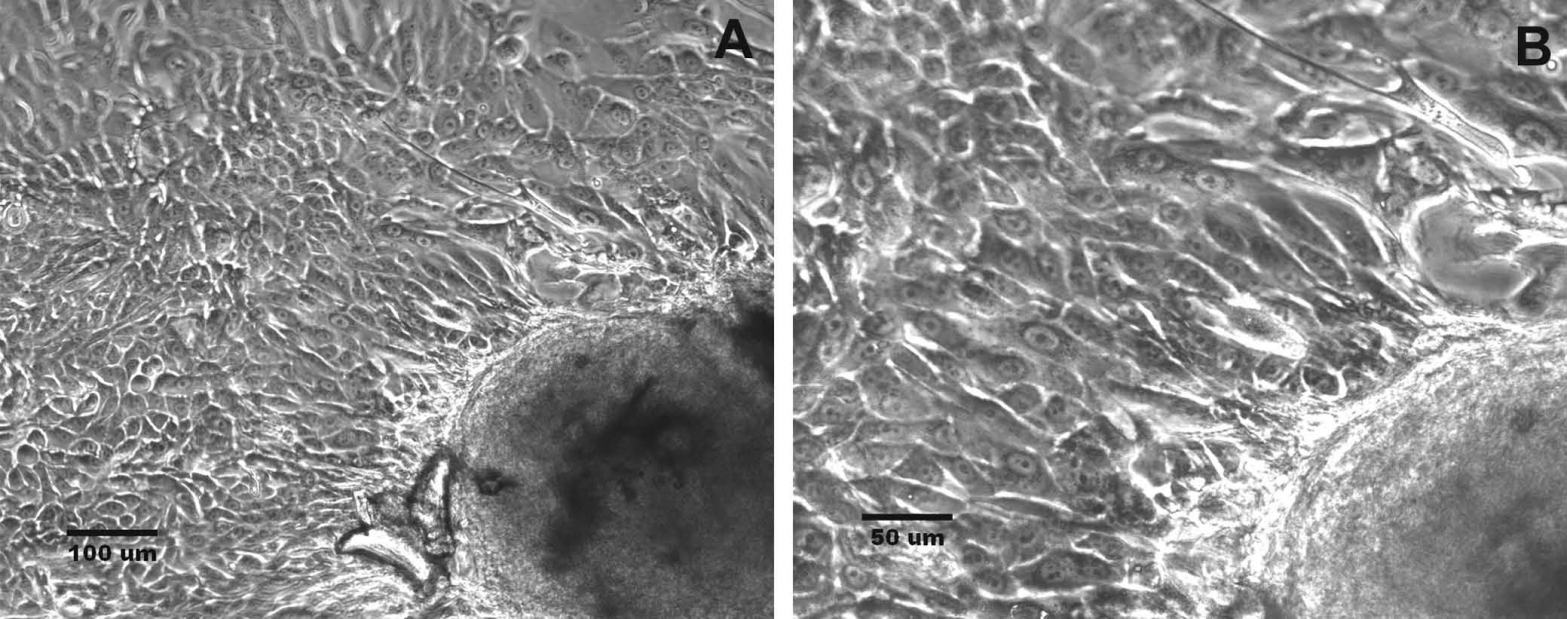
**Morphologic appearance of early tumor explants from HNSCC biopsy samples**. Phase-contrast photomicrograph of tumor explants isolated from HNSCC biopsies in culture after 2-4 weeks with cells growing directly from the explants in a monolayer (A ×100, B ×200 original magnification).

**Figure 4:**
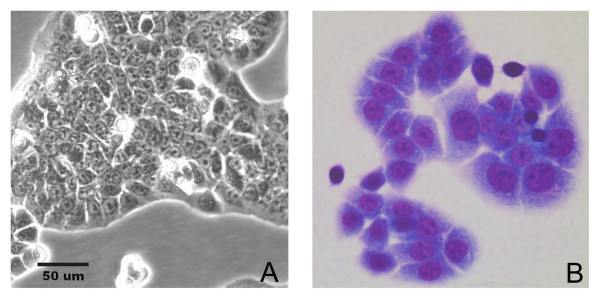
**Establishment of the USC-HN1 cell line**. (A) Phase contrast microphotograph of growing USC-HN1 cells showing numerous mitotic cells (rounded, luminescent cells) with a tightly, adherent squamous cell morphology. (B) Cytology of the USC-HN1 cell line shows malignant cells with large nuclei and nucleoli and an abundance of cytoplasm typical of squamous cells (Cytospin, Wright-Giemsa stain, ×100 original magnification).

A cell line, designated USC-HN1, was established from the primary tumor biopsy specimen after approximately 4 weeks in culture. Initially, explants were seen attaching to the flask and tumor cells grew centrifugally from these small tissue fragments (Figure 3). By ten days the explants were trypsinized and re-plated as single cells along with growing stromal cells. At this time, explants were grown only in Pen/Strep antibiotic solution (Gibco, Carlsbad, CA) and were not affected by the early use of anti-mycotic or other antibacterial reagents that were added to the early cultures. By 3 weeks, tumor cells were seen rounding up and growing throughout the T-75 flask amongst the normal stromal fibroblasts that grew in parallel. Differential trypsinization was used to remove the majority of the fibroblasts which detached sooner than the tumor cells. To isolate a pure population of tumor cells, 100 detached cells were plated in 100 mm petri dishes and colonies were removed by trypsinization using cloning rings. These isolated colonies were used to establish the cell line which typically grows rapidly forming tight monolayers in flasks. The cells are easily detached with pre-warmed 0.05% trypsin/EDTA (Gibco) and were found to have a rapid doubling time of 18 hr. A search of the literature shows that other head and neck squamous cell lines have doubling times between 17-240 hrs with a median time of 26.5 hr [1]. In culture, the USC-HN1 cell line has numerous mitotic figures, is tightly adherent, and demonstrates typical squamous cell morphology (Figure 4A) that has remained constant since the establishment of the cell line. Wright-Giemsa staining of cell-cultured slide preparations shows malignant cells with large nucleoli, some cytoplasmic vacuoles, and an abundance of cytoplasm typical of squamous cells (Figure 4B). Mycoplasma testing on multiple passages was found to be negative.

### Heterotransplantation into Nude mice

**Figure 5:**
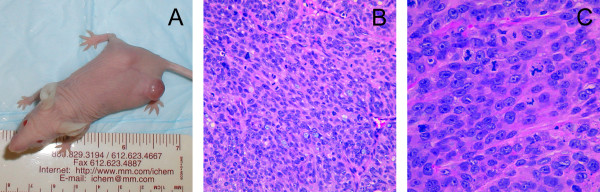
**Heterotransplantation of USC-HN1 cell line into Nude mice**. (A) Appearance of subcutaneous USC-HN1 tumor in Nude mouse. (B and C) Low and high magnification of USC-HN1 Nude mouse heterotransplant showing a poorly differentiated squamous cell carcinoma arranged as a sheet with areas of tumor necrosis and bluntly infiltrative borders. The tumor cells are tightly cohesive featuring from moderate to abundant eosinophilic cytoplasm. The nuclear to cytoplasmic ratio is markedly increased with nuclei showing frequent, prominent nucleoli. Mitotic activity is abundant including atypical forms such as ring and tripolar configurations. Intercellular bridges are focally present but faint (H&E ×200 and ×400 original magnification).

Cultured USC-HN1 cells were heterotransplanted subcutaneously in Nude mice to produce expanding tumors in 5/5 mice that displayed the cell line's malignancy (Figure 5). At 3 weeks, the tumors were excised. Histologic sections of the heterotransplanted tumor stained with hematoxilin and eosin show a poorly differentiated squamous cell carcinoma arranged as a sheet with areas of tumor necrosis and bluntly infiltrative borders. The tumor cells are tightly cohesive featuring from moderate to abundant eosinophilic cytoplasm. The nuclear to cytoplasmic ratio is markedly increased with nuclei showing frequent, prominent nucleoli. Mitotic activity is abundant including atypical forms such as ring and tripolar configurations. Intercellular bridges are focally present but faint.

### Immunophenotype of USC-HN1 in cell culture and in situ

**Figure 6:**
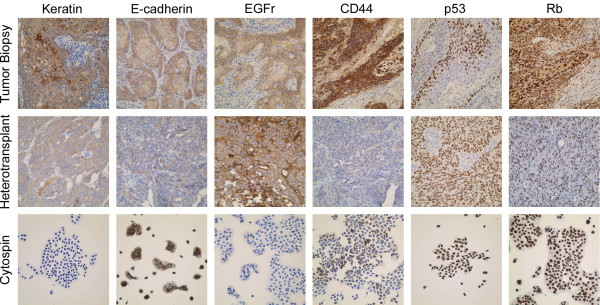
**Immunoperoxidase staining of USC-HN1 cells in Nude mouse heterotransplant and in cytospin preparations for HNSCC classification markers**. Photomicrograph of immunoperoxidase staining of original tumor biopsy (top), IHC stained formalin-fixed paraffin-embedded tissue sections of USC-HN1 Nude mouse heterotransplant (middle), and USC-HN1 cells from culture in a cytospin preparation (bottom) for keratin, E-cadherin, EGFr, CD44, p53 and Rb (×200 original magnification).

Immunophenotypic characterization of USC-HN1 cells in culture and from tumors grown in Nude mice demonstrated similarity to the original tumor and confirmed a keratinizing squamous cell carcinoma (Figure 6). USC-HN1 cells demonstrate strong, uniform nuclear p53 and Rb expression, as well as positive expression of keratin, E-cadherin, EGFr, and CD44 *in situ *consistent with HNSCC as well as the original tumor biopsy [4,7,13,19]. There is decreased staining of E-cadherin and CD44 *in situ *compared with cultured cells. Conversely, EGFr staining is stronger in those cells grown as heterotransplants. CD44 staining is decreased in the heterotransplant and cytospin in comparison with the original tumor biopsy.

**Figure 7:**
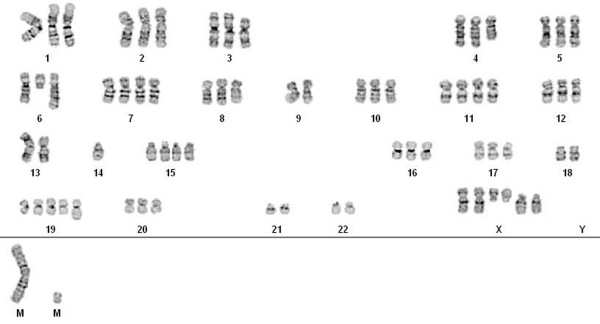
**Karyotype of USC-HN1**. Karyotype of USC-HN1 cell line showing a near-triploid clone (modal 71) demonstrating features characteristic of head and neck cancer including multiple deletions (chromosomes X, 3, 4, 6, and 7), isochromosome formation, and many breakpoints around centromeres.

### USC-HN1 characterization by flow cytometry

The phenotype of USC-HN1 cell line was further characterized by surface and intracellular staining for common HNSCC biomarkers by fluorescently-labeled monoclonal antibodies and analysis by flow cytometry (Table 2). Compared to isotype controls, USC-HN1 displays significantly increased staining of FABP5, E-cadherin, and CD24. EGFr, *c-Kit*, and CD74 staining also showed an increased mean fluorescence intensity, whereas IL-13Rα, CD44v6 and CD133 did not show a significant increase.

**Table 2:** Analysis of USC-HN1 cytokines and surface markers by FACS.

	% Positive		MFI	
Target	Isotype Control	Antibody	Isotype Control	Antibody
FABP5	0.01	97.49	31.80	1310.47 ***
E-cadherin	0.01	49.74	6.87	203.69 ***
CD24	0.01	7.87	6.87	90.52 ***
EGFR	4.50	10.71	37.70	198.78 **
*c-Kit*	0.01	0.26	6.55	14.01 **
CD74	0.01	0.15	6.87	9.16 **
IL-13Rα	5.47	2.63	8.89	7.89 *
CD44v6	0.06	0.04	13.27	9.38 *
CD133	7.57	3.02	38.84	20.67 *

*** > 1000% Control MFI

** 100-1000% Control MFI

* < 100% Control MFI

USC-HN1 cells in culture were stained for HNSCC-related cytokines and surface markers (left column) using monoclonal antibodies and analyzed by flow cytometry. Samples were run in duplicate. Percent of positive staining cells (middle columns) and mean fluorescence intensity (right columns) is shown for each antibody target and its isotype control.

### Cytogenetic analysis

Cytogenetic analysis of the USC-HN1 cell line was performed in collaboration with the City of Hope (Duarte, CA) (Figure 7). All mitotic cells analyzed from the USC-HN1 cell line were clonally abnormal. The complex near-triploid clone was characterized by a modal number of chromosomes of 71 and by deletions involving chromosomes X, 3, 4, 6, and 7, additional material of unknown origin on chromosomes 8, 13, 17, and 19, a derivative chromosome 6 resulting from an unbalanced translocation with the long arm of chromosome 14, gains of chromosomes X, 7, 11, 15, and 19, losses of chromosomes 9, 14, 18, 21, and 22, as well as gain of a large marker chromosome and a small suspected ring chromosome.

### PCR Viral screen for HPV and EBV

**Figure 8:**
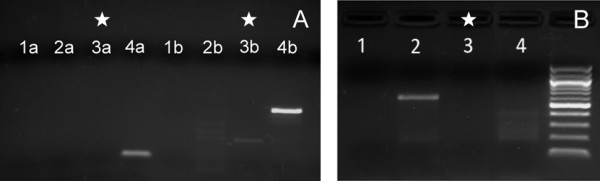
**PCR studies for the presence of oncogeneic viruses**. (A) HPV detection using consensus primers GP5^+^/GP6^+ ^(1a-4a) and MY09/MY11 (1b-4b) demonstrating USC-HN1 (3a, 3b) is negative for HPV infection. Positive (HeLa 4a, 4b), negative (SW579 2a, 2b), and water (1a, 1b) controls were run in parallel. (B) PCR study for detection of EBV with consensus primer showing USC-HN1 negative (3). Positive (Raji, 2), negative (HUT102, 4), and water (1) controls were run in parallel.

Since HPV and EBV are associated with malignancies of the head and neck, the USC-HN1 cell line was screened for these oncogenic viruses by PCR using consensus primers for HPV L1 and EBV EBNA2 genes as described previously [15,17]. The results showed that the USC-HN1 did not have specific amplifications for either the HPV L1 sequence or for the EBV sequence (Figure 8). HeLa cells (known HPV-positive cervical carcinoma) and SW579 (known HPV-negative thyroid carcinoma) were run in parallel as controls for both HPV consensus primer sets (MY09/MY11 and GP5+/GP6+). For the EBV screen, EBV^+ ^Raji and EBV^- ^T-cell lymphoma TLBR-1 cell lines [17,20] were run in parallel as controls.

### Cytokine and oncogenes expression

The cell line was also tested by qRT-PCR for its expression levels of pertinent oncogenes and cytokines. Run in parallel with RNA extracted from the human pharyngeal carcinoma FaDu cell line and compared to Universal Human Reference RNA, the overall expression levels of USC-HN1 closely paralleled that of FaDu, a documented HNSCC cell line (Table 3). Statistical analysis revealed significant differences between the expression levels of *c-myc*, VEGFa, TGF-β1, and IL-1β produced by USC-HN1 and the FaDu cell line, and no significant differences between p53, Rb, *c-Kit*, VEGFc, COX2, TGF-β2, IL-4, IL-6, IL-8, and IL-10.

**Table 3:** Oncogene and cytokine analysis of HNSCC cell lines by qRT-PCR.

	USC-HN1	FaDu	
Gene	Average Fold Δ	Average Fold Δ	t-test
p53	0.2111	0.3671	0.5377
Rb	1.1222	0.5076	0.2022
***c-myc***	**0.9730**	**0.5848**	**0.0025**
***c-Kit***	**0.0006**	**0.0002**	**0.0286**
			
**VEGFa**	**0.4272**	**0.1406**	**0.0026**
VEGFc	0.2402	0.1450	0.7093
COX2	0.0385	0.0064	0.4963
**TGF-B1**	**1.1887**	**0.1312**	**0.0205**
TGF-B2	0.0261	0.0172	0.2514
**IL-1B**	**0.2091**	**29.4400**	**0.0064**
IL-4	0.3472	0.0767	0.0660
IL-6	0.0661	0.0905	0.1403
IL-8	0.1866	0.1293	0.1389
IL-10	0.0204	0.0078	0.3217

RNA was extracted from USC-HN1 and FaDu cell lines, treated with DNase, and analyzed against Universal Human Reference RNA. Gene amplification was normalized to GAPDH. Analysis of oncogenes and cytokines depict an overall similar profile for both USC-HN1 and FaDu. *C-myc*, *c-Kit*, VEGFa, and TGF-B1 are significantly upregulated in USC-HN1 compared to FaDu; IL-1B was significantly down-regulated.

### Activated *Notch1 *analysis

**Figure 9:**
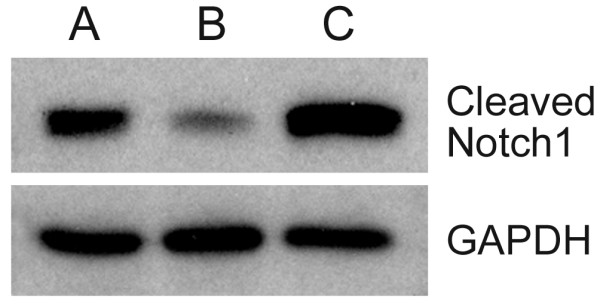
**Western Blot: *Notch1***. Western blot analysis of the active, cleaved portion of *Notch1 *run in parallel with confirmatory GAPDH. (lane A) Karpas 299 positive control; (lane B) Siha low expression control; and (lane C) USC-HN1 shows high expression levels of *Notch1 *protein.

*Notch1 *protein levels were determined by Western blot for the USC-HN1 cell line in comparison to expression levels seen in positive (Karpas 299) and negative (Siha) cell line controls. Based upon these studies, USC-HN1 demonstrated an increased level of activated *Notch1 *protein at levels equal to or higher than the Karpas 299 positive control (Figure 9).

### Microarray gene expression profiling

Microarray gene expression analysis was performed on isolated total RNA from USC-HN1 cells and compared to the results previously reported in HNSCC tumor biopsy samples [6]. To compare the similarities of gene expression profile between the USC-HN1 cells and HNSCC tumor biopsy samples, we focused on the up-regulated genes in USC-HN1 cells. A total of 328 genes with *p *< 0.05 and log2 fold ≥ 1 in USC-HN1 cells compared with previous results from normal tonsils were identified. Many up-regulated genes identified in USC-HN1 cells were also present in HNSCC biopsies (Table 4). These common genes were classified into various categories of biological functions including those related to immune response such as MIF and CD24, which were currently identified in HNSCC [6]. Other signatures of up-regulated genes in USC-HN1 cells include ATP5H, HSP27, FABP5, E-cadherin, EIF4G2, KRT18 and KRT8, RPLP0 and RPS18, which are associated with various biological processes such as cell growth and maintenance, cell cycle regulation, metabolism, and protein translation and synthesis (Table 4).

**Table 4:** Selected up-regulated genes identified in USC-HN1 cells by microarray analysis also present in HNSCC tumor biopsies.*

GeneBank Access ID	Gene Symbol & Annotation	Log2 Fold Difference**
***Immune Response***		
L19686	MIF, macrophage migration inhibitory factor	3.1
NM_002116	HLA-A, major histocompatibility complex, class I A	2.2
X16302	IGFBP2, insulin-like growth factor binding protein 2	1.7
L33930	CD24, CD24 antigen	1.1
		
***Cell Growth, Maintenance/Cell cycle Regulation***		
M26326	KRT18, keratin 18	6.2
NM_002273	KRT8, keratin 8	5.8
X95404	CFL1, cofilin 1	4.1
NM_005022	PFN1, profilin 1	4.0
Y00503	KRT19, keratin 19	3.6
AL031670	FTL, ferritin, light polypeptide	3.2
AF026291	CCT4, chaperonin subunit 4	3.2
NM_004360	CDH1, E-cadherin	2.3
		
***Translation and Protein Synthesis***		
AK001313	RPLP0, ribosomal protein LP0	6.3
L11566	RPL18, ribosomal protein L18	5.5
M64241	RPL10, ribosomal protein L10	5.2
X69150	RPS18, ribosomal protein S18	4.9
U73824	EIF4G2, translation initiation factor 4 gamma 2	4.2
L06499	RPL37A, ribosomal protein L37A	4.0
M84711	RPS 3A, ribosomal protein S3A	3.8
U25789	RPL21, ribosomal protein L21	3.7
AL117412	EIF4A2, eukaryotic translation initiation factor 4A	2.9
Z21507	EEF1D, eukaryotic translation elongation factor 1D	1.2
		
***Metabolism***		
M26252	PKM2, pyruvate kinase, muscle	5.7
NM_021130	PPIA, peptidylprolyl isomerase A (cyclophilin A)	4.7
X13794	LDHB, lactate dehydrogenase B	4.4
Z23090	HSPB1, heat shock 27 kDa protein	4.2
Y13936	PPM1G, protein phosphatase 1G	2.8
U09813	ATP5G3, ATP synthase H+ transporting subunit	2.7
AF061735	ATP5H, ATP synthase H+ transporting subunit	2.5
D29011	PSMB5, proteasome subunit, beta 5	2.4
Y00483	GPX1, glutathione peroxidase 1	2.3
M60483	PPP2CA, protein phosphatase 2 catalytic subunit	2.2
NM_001679	ATP1B3, ATP synthase Na+/K+ transporting, beta 3	1.6
M94856	FABP5, fat acid binding protein 5	1.2
		
***Ion Binding Proteins***		
D38583	S100A11, S100 calcium binding protein A11	1.8
NM_020672	S100A14, S100 calcium binding protein A14	1.0
		
***Others***		
S54005	TMSB10, thymosin, beta 10	4.2
M14328	ENO1, enolase 1	4.0
M26880	UBC, ubiquitin C	3.4
X67951	PRDX1, peroxiredoxin 1	3.2
M36981	NME2, non-metastatic cells 2 protein	3.1
NM_006016	CD164, CD164 antigen	2.6

*All selected genes with *p*-value < 0.05.

**Log2 ratio fold-differences between USC-HN1 cells and normal tonsil tissues were determined by subtracting the ratio of genes in normal tonsil tissue versus uRNA from the ratio of genes in USC-HN1 cells versus uRNA. (Log2 ratio of 1 equals to 2-fold difference between USC-HN1 cells and normal tissue; Log2 ratio of 2 equals to 4-fold difference between USC-HN1 and normal tissue, and so on).

## Discussion

We describe the clinical presentation of an invasive well-differentiated maxillary HNSCC and the establishment of the USC-HN1 cell line from the primary tumor biopsy. Of note, patient NR was a non-smoker and did not receive preoperative radiotherapy or chemotherapy. Her tumor was consistent with a diagnosis of primary HNSCC on presentation by tissue histology and immunostaining for HNSCC markers. The USC-HN1 cell line was established in culture approximately 4 weeks after seeding by isolation from co-existing fibroblastic monolayers which grew from the explanted cultures. USC-HN1 shares many characteristics with the primary tumor and has a phenotype typical of an advanced HNSCC. The doubling time of the cell line is extremely rapid (18 hr) compared to other human tumor cell lines from HNSCC [1] or those from other tumor types.

To demonstrate its malignant potential, the cell line was successfully heterotransplanted into Nude mice where it was found to produce invasive tumors. In comparison to the original tumor, the heterotransplant most prominently shows a higher nuclear to cytoplasmic ratio, markedly increased mitotic activity, prominent necrosis and less conspicuous intercellular bridging. Marked keratinization is also absent. The difference between the original tumor histology and the subsequently developed cell line's heterotransplant is likely due to the outgrowth of a more aggressive, less differentiated cell sub-population during establishment of the cell line in culture. In addition, sections of the heterotransplanted tumors stained for HNSCC markers were mostly consistent with previously reported primary tumor biopsies including strong EGFr, p53, and Rb staining, as is seen in many epithelial malignancies [4]. Furthermore, fixed sections of the original tumor biopsy and cytospin cell preparations were also stained for these biomarkers. Noted differences of the cytospin preparations relative to the heterotransplanted tumors were a decrease in keratin and EFGr staining and an increase in E-cadherin and CD44 positivity. The moderate staining of keratin found in the heterotransplant tumor sections is similar with the original tumor, described as a keratinizing squamous cell carcinoma. The light staining of E-cadherin in the heterotransplanted tumor may indicate the malignant potential of the cells as they begin to undergo epithelial-to-mesenchymal transition and consequently lose cell-cell adhesive and interactive molecules [12,19]. When the malignant cells are placed in a murine host, they are subjected to a myriad of microenvironmental factors different from that of cell culture medium or a human host. The loss of CD44 in the heterotransplanted tumor sections may be explained by the presence of factors that increase the differentiation of USC-HN1 *in situ *in contrast to its stem cell-like phenotype that predominates in cell culture or *in vivo *[8].

Analysis of the flow cytometry data supports a comparable picture to the IHC in which almost all USC-HN1 cells were positive for FABP5 and half were E-cadherin positive (97.5% and 49.7%, respectively). Furthermore, USC-HN1 showed a significant increase in EGFr staining as is to be expected in an epithelial malignancy [4]. Interestingly, stem cell markers were not uniformly expressed in the sample of cultured HNSCC although CD24 and CD74 showed increased staining unlike CD133. USC-HN1 is grown in a serum-containing medium, which may irreversibly differentiate any stem cells in the population and therefore alter the surface marker expression in the remaining pluripotent cells [8]. Conversely, these cells may represent only a subpopulation of tumor stem cells found previously to display various combinations of CD24, CD74, and CD133 [2]. Our results did not show a significant amount of surface staining for the recently published markers IL-13Rα or CD44v6. However, these published studies were performed on primary tumor biopsies and not on established cell lines [6]. The karyotype analysis of USC-HN1 did show a highly abnormal chromosome content consistent with other known HNSCC cell lines and solid tumors. Aneusomy is a recurrent finding in cytogenetically abnormal head and neck tumor specimens [9]. Deletions are the most common structural rearrangement observed, particularly involving the short arms of chromosomes 3 and 8, as well as the short arm regions of the acrocentric chromosomes. Many structural breakpoints in HNSCC involve centromeric or pericentromeric regions of the chromosomes, often resulting in isochromosome formation or derivative whole-arm translocations involving the acrocentric chromosomes [Personal communication with Dr. Joyce L. Murata-Collins, Division of Anatomic Pathology, City of Hope, Duarte, CA]. Most notably, the partial deletion of chromosome 3 and the near-triploid karyotype of the clonal population are consistent with the HNSCC-derived nature of this cell line [9]. Viral oncogene investigation detected no infection with either HPV or EBV. In this regard, 80-90% of nasopharyngeal carcinomas have been shown to be infected with EBV but very few (<1%) of HNSCC are positive. An extensive literature report revealed only two cases of EBV^+ ^HNSCC and both cases were co-infected with HPV [21,22]. The EBV-negative characteristic of USC-HN1 further supports the origin of the tumor as a squamous and not a nasopharyngeal carcinoma. By contrast, 30% of HNSCC are infected with a subtype of HPV [12]. USC-HN1 represents a unique HPV-negative HNSCC cell line in that it was derived from a patient who did not smoke tobacco or drink alcohol and does not have a family history of head and neck malignancies.

Further characterization of the USC-HN1 cell line revealed a similar pattern of cytokine and chemokine expression compared to the pharyngeal carcinoma FaDu, a widely used HNSCC cell line. As previously reported HNSCC are highly immunomodulatory and alter their tumor microenvironment by the production of various cytokines [3]. The production of VEGFc has been correlated with increased metastatic potential in HNSCC [23], and USC-HN1 has shown a statistically increased production of VEGFc compared with FaDu. Importantly, with the exception of *c-myc*, the USC-HN1 cell line reveals statistically equivalent expression of proto-oncogenes and tumor-suppressor genes as FaDu and a cytokine expression profile different from FaDu, which will offer researchers yet another biomodel to study the microenvironment of HNSCC. Finally, the increased levels of activated, cleaved *Notch1 *found in USC-HN1 are indicative of its malignant potential, and although published reports imply various levels of *Notch1 *activity among HNSCC cell lines [24], it may serve as a future avenue for a new therapeutic approach since multiple trials of *Notch1 *inhibitors are in progress in patients with other tumor types [14].

Microarray analysis of USC-HN1 revealed a pattern of gene expression similar to HNSCC tumor samples previously reported [6]. In addition, specific up-regulated genes including CD24, E-cadherin, FABP5, keratins, heat shock protein (27 kDa), and others identified by microarray analysis were also identified by flow cytometry and immunostaining. These results support the origin of the cell line and further confirm the pathology as HNSCC.

## Conclusions

In conclusion, we have established a novel HPV-negative HNSCC cell line from a non-smoking elderly patient. USC-HN1, one of only a few cell lines derived from the upper alveolar ridge, recapitulates the primary tumor's malignant behavior. It also displays surface and intracytoplasmic biomarkers consistent with HNSCC. This cell line will provide researchers with a well-delineated model to investigate the oncogenesis of HNSCC and may provide a source of material to develop new therapeutic reagents capable of treating these deep-seated and highly resistant tumors.

## Competing interests

The authors declare that they have no competing interests.

## Authors' contributions

Contribution: DJL characterized the USC-HN1 cell line, analyzed the data and wrote the paper; MGL assisted with the heterotransplantation studies, flow cytometry, and western blots; RM assisted with transfer of the cell line and provided cell culture expertise; UKS identified the patient and excised the original tumor biopsy; JH and RKP performed the microarray experiments, data analysis and interpreted results and composed the corresponding sections; AJC reviewed the histological slides and provided pathology expertise; ALE designed the study, established the cell line, and supervised the studies. All authors reviewed the paper.
